# Cost-effectiveness of a coronary heart disease secondary prevention program in patients with myocardial infarction: results from a randomised controlled trial (ProActive Heart)

**DOI:** 10.1186/1471-2261-13-33

**Published:** 2013-05-01

**Authors:** Erika Turkstra, Anna L Hawkes, Brian Oldenburg, Paul A Scuffham

**Affiliations:** 1 Centre for Applied Health Economics, School of Medicine, Griffith Health Institute, Griffith University, Brisbane, QLD, Australia; 2 School of Public Health and Social Work, Queensland University of Technology, Brisbane, Australia; 3 School of Public Health and Preventive Medicine, Monash University, Melbourne, Australia

## Abstract

**Background:**

Participation in coronary heart disease (CHD) secondary prevention programs is low. Telephone-delivered CHD secondary prevention programs may overcome the treatment gap. The telephone-based health coaching ProActive Heart trial intervention has previously been shown to be effective for improving health-related quality of life, physical activity, body mass index, diet, alcohol intake and anxiety. As a secondary aim, the current study evaluated the cost-effectiveness of the ProActive Heart intervention compared to usual care.

**Methods:**

430 adult myocardial infarction patients were randomised to a six-month CHD secondary prevention ‘health coaching’ intervention or ‘usual care’ control group. Primary outcome variables were health-related quality of life (SF-36) and physical activity (Active Australia Survey). Data were collected at baseline, six-months (post-intervention) and 12 months (six-months post-intervention completion) for longer term effects. Cost-effectiveness data [health utility (SF-6D) and health care utilisation] were collected using self-reported (general practitioner, specialist, other health professionals, health services, and medication) and claims data (hospitalisation rates). Intervention effects are presented as mean differences (95% CI), p-value.

**Results:**

Improvements in health status (SF-6D) were observed in both groups, with no significant difference between the groups at six [0.012 (-0.016, 0.041), p = 0.372] or 12 months [0.011 (-0.028, 0.051) p = 0.738]. Patients in the health coaching group were significantly more likely to be admitted to hospital due to causes unrelated to cardiovascular disease (p = 0.042). The overall cost for the health coaching group was higher ($10,574 vs. $8,534, p = 0.021), mainly due to higher hospitalisation (both CHD and non-CHD) costs ($6,841 vs. $4,984, p = 0.036). The incremental cost-effectiveness ratio was $85,423 per QALY.

**Conclusions:**

There was no intervention effect measured using the SF-36/SF-6D and ProActive Heart resulted in significantly increased costs. The cost per QALY gained from ProActive Heart was high and above acceptable limits compared to usual care.

## Background

Coronary heart disease (CHD) is a major cause of morbidity, mortality and economic burden in Australia and the rest of the developed world [1]. Secondary prevention programs, with a focus on risk factor management, have been shown to play a pivotal role in the treatment and management of those affected by CHD. The clinical benefits of secondary prevention / cardiac rehabilitation programs include decreased total cardiac mortality (26%), improved health-related quality of life (HRQoL), and lower rates of rehospitalisation [2-4]. As such, guidelines recommend that all persons with CHD participate in secondary prevention programs [5,6].

Telephone-delivered interventions are one option to deliver cardiac rehabilitation [7-9] and there is a large body of literature on home-based and telehealth programs for patients with heart failure [10] or diabetes [11]. While there is a growing body of evidence demonstrating the potential benefits of telephone interventions for cardiac patients there is a paucity of evidence on the cost-effectiveness of telephone-based interventions [12]. A recent systematic review by Goode *et al.* (2012) identified two publications on the cost-effectiveness of telephone-delivered interventions using physical activity and dietary behaviour change [12]. Both publications supported the cost-effectiveness of telephone-delivered interventions [13,14], albeit the interventions and populations were different from that in the ProActive Heart study. In the randomised controlled trial reported by Graves *et al.* (2009) the trial-based intervention was not compared with the usual care (UC) group but used a theoretical UC group instead [14]. A systematic review that included *costs* as a key outcome identified 28 tele-rehabilitation studies. That review reported that there was preliminary evidence around potential cost-savings to the health system and concluded that “high-quality evidence regarding impact on resource allocation and costs is still needed to support clinical and policy decision-making” [15].

The ProActive Heart trial was a novel telephone-delivered secondary prevention program. The primary aim of that study was to achieve significantly greater improvements in HRQoL and physical activity for a health coaching (HC) intervention versus UC patients. The efficacy outcomes have been published previously [16,17]; in summary, significant intervention effects were observed for mental HRQoL (*p* = 0.02), social functioning (*p* = 0.04) and role-emotional (*p* = 0.03) subscales compared with UC. Intervention participants were also more likely to meet recommended levels of physical activity (*p* = 0.02), body mass index or BMI (*p* = 0.05), vegetable intake (*p* = 0.04), and alcohol consumption (*p* = 0.05) [16]. A significant intervention effect was also observed for anxiety compared with UC (*p* = 0.04) [17]. A secondary aim of the study was to examine the cost-effectiveness of the ProActive Heart intervention, which we report here.

## Methods

The study design, aims and recruitment procedures have been published previously [18]. In brief, patients with a recent myocardial infarction (MI) were randomised to the HC or UC group. Participants in both groups completed assessments at baseline, post-intervention or six-months follow-up, and at 12-months follow-up. The cost-effectiveness results are based on six-months of follow-up data as this was the primary end point. Ethics approval was received from Human Research Ethics Committees of The Prince Charles Hospital (EC2738), the Royal Brisbane and Women’s Hospital (2007/049), and Monash University (2007/0584MC). We recruited 430 adult MI patients over a 14 month period (December 2007 to January 2009) from two large metropolitan hospitals in Brisbane, Australia (Royal Brisbane and Women’s, and The Prince Charles Hospitals).

### Study conditions

The intervention commenced within the first two weeks of discharge. UC participants received existing written educational resources. Over a six-month period, HC participants received 10 × 30 minute scripted telephone HC sessions from a qualified health professional or ‘health coach’. Details of the telephone HC sessions have been provided previously [18].

### Measurement

Clinical data were collected at baseline from hospital medical records. Socio-demographic data was self-reported at baseline and additional data were collected at baseline, six and 12 months by computer assisted telephone interview (CATI).

### Cost-effectiveness

A cost-effectiveness analysis of the costs and outcomes for patients in the intervention and control groups was conducted from the perspective of health care costs to the Australian government. The primary health outcome for the cost-effectiveness analyses was quality-adjusted life years (QALYs). These were calculated for both groups using HRQoL scores from the SF-36 [19] and converted to utility scores using the SF-6D, using UK weights [20].

### Resource use and costs

Details of the resource use and costs are presented in the Web Appendix Additional file 1: Table S1. In summary, most of the resource utilisation was based on self-reporting [general practitioner (GP) visits, specialist visits, other health professionals visits, and medication]. Hospital resource use and inpatient costs using AR-DRGs were sourced from Queensland Health Admitted Patient Data Collection which includes all public hospital separations in Queensland. Hospitalisation was categorised by cardiovascular hospitalisations (including MI, angina and chronic heart failure) and other hospitalisations. The GP visits were validated from a randomised sub-sample of 10% of patient records and provider surveys, which indicated underreporting. To adjust for this underreporting a correlation coefficient was applied to the patient reported GP visits to estimate the actual visits (See Additional file 1).

National average costs for each item of resource use were applied (Additional file 1). All costs are reported in 2008 Australian dollars.

### Data analysis

To assess differences in baseline characteristics between HC and UC groups Pearson chi-squared tests were used for categorical data and Student’s t-tests were used for continuous data. As there was missing data for the SF-6D at six months (HC n = 73, UC n = 62 missing), multiple imputation methodology was applied to the data, using gender and baseline SF-6D values as predictors. For the resource use data, multiple imputation methodology was applied, using gender as a predictor. The inclusion of gender as a predictor is justified, as the trial was stratified by gender, and availability of data at six months was dependent on gender, with more males reporting at six month (73.5%) compared to females (58.7%; Fisher’s Exact Test p = 0.005). Data on SF-6D, resource use and cost were analysed using the independent samples Mann–Whitney U test. Statistical significance was determined using p-value <0.05. All statistical analyses were performed using SPSS (version 19.0).

## Results

### Baseline characteristics

The flow of the participants and detailed baseline characteristics have been previously published [16]. A summary of the patient baseline characteristics is presented in Table 1, and as can be observed, characteristics were similar between the HC and UC group.

**Table 1:** Baseline characteristics

**N**	**HC 215**	**UC 215**	***P-value***
Age, year	61.3 (11.3)	59.9 (11.1)	0.212
Male,%	75.8%	73.5%	0.579
Smoking status			
• Never smoked	25.1%	30.2%	0.398
• Previous smoker	43.7%	38.1%	
• Current smoker	31.1%	31.6%	
Doctor visits in last six months	4.55 (4.18)	4.79 (4.24)	0.588
Number of times admitted to hospital in last six months	1.99 (0.95)	1.97 (0.95)	0.840

Overall, 83% of all participants randomised to receive HC received at least 5 of 10 possible telephone sessions. The median number of sessions was 8 (range 0–10) and the mean (SD) call length was 26 (9.3) minutes.

### Health status

Using the SF-6D, there were no statistically significant differences in baseline health status or utility between the HC and UC groups (Table 2). At six months, SF-6D data was available for 141/215 (65.6%) of the HC group and 153/215 (71.2%) of the UC group (See Web Appendix Additional file 1: Table S2). Using multiple imputation an increase in health utility was observed in both groups [HC: 0.130 (95% CI: 0.111 to 0.149); UC: 0.118 (95% CI: 0.097 to 0.139); Table 2] and no differences were observed between the two groups (p = 0.372). At 12 months, no statistically significant further improvement in SF-6D was observed in either group, resulting in an incremental from baseline of 0.132 (95% CI: 0.110 to 0.153) for HC and 0.120 (95% CI: 0.098 to 0.142) for UC. Analyses using completed cases only resulted in similar results (See Web Appendix Additional file 1: Table S3).

**Table 2:** **SF-6D index at baseline and change between health coaching (HC) and usual care UC**^**a**^

**Mean (SE)**	**HC Mean (SE)**	**UC Mean (SE)**	**Mean difference (95% CI)**	***p-value***
N	215	215		
Baseline	0.680 (0.009)	0.675 (0.009)	0.005 (-0.021, 0.031)	0.739
Change from baseline - 6 months^b^	0.130 (0.010)	0.118 (0.011)	0.012 (-0.016, 0.041)	0.372
Change from baseline - 12 months^b^	0.132 (0.011)	0.120 (0.011)	0.011 (-0.028, 0.051)	0.738

^a^ Using multiple imputation techniques where gender was used as a predictor.

^b^ Adjusted for baseline value.

### Health care utilisation

The main difference in health care utilisation was that HC participants were statistically significantly more likely to be hospitalised compared to UC patients (Table 3). *‘This difference was mainly due to non-cardiovascular hospitalisations’*. The main non-cardiovascular hospitalisations for patients in the HC group were urinary and renal disease (including dialysis), cancer, and gastric disorders, while for the education group the main non-cardiovascular hospitalisations and costs were for urinary and renal disease (including dialysis) and cancer (data not provided). A similar pattern was observed when the analysis was performed for completed cases only (See Web Appendix Additional file 1: Table S4).

**Table 3:** **Utilisation and cost of health care services for health coaching (HC) and usual care (HC) groups **^**a**^

	**Utilisation**			**Cost**		
**Mean (se)**	**HC**	**UC**	**p-value**	**HC**	**UC**	**p-value**
N	215	215		215	215	
Health coach sessions	7.2 (0.2)		N/A	$267 ($7)	-	N/A
General Practitioner visits	11.2 (0.4)	11.8 (0.3)	0.188	$446 ($14)	$470 ($14)	0.188
Specialist visits	1.6 (0.2)	1.1 (0.1)	**0.004**	$88 ($7)	$61 ($5)	**0.004**
Other health professionals	2.2 (0.3)	1.6 (0.2)	**0.048**	$104 ($10)	$81 ($8)	**0.048**
Health services	4.8 (0.4)	5.1 (0.5)	0.815	$1,161 ($101)	$1,222 ($119)	0.798
Medication						
• Cardiac system	2.4 (0.1)	2.5 (0.1)	0.414	$312 ($16)	$329 ($16)	0.328
• Lipid modifying drugs	0.8 (0.0)	0.9 (0.1)	0.486	$474 ($17)	$492 ($16)	0.616
• Antithrombotic agents	1.6 (0.0)	1.7 (0.0)	0.462	$385 ($14)	$394 ($15)	0.593
• Drugs used in diabetes	0.4 (0.0)	0.4 (0.0)	0.365	$191 ($29)	$169 ($27)	0.419
• Other medicines	1.4 (0.1)	1.3 (0.1)	0.593	$305 ($26)	$332 ($52)	0.536
Hospital admittance/patient						
• MI/angina/CHF	0.6 (0.1)	0.5 (0.1)	0.240	$4,714 ($641)	$4,139 ($771)	0.240
• Other causes	0.9 (0.5)	0.2 (0.1)	0.054	$2,127 ($558)	$846 ($214)	**0.043**
• Total	1.5 (0.5)	0.7 (0.1)	**0.042**	$6,841 ($838)	$4,984 ($802)	**0.036**
Total cost				$10,574 ($855)	$8,534 ($813)	**0.021**

^a^ Using multiple imputation techniques where gender was used as a predictor.

MI, myocardial infarction; CHF, chronic heart failure.

### Costs

The major difference in costs between the two groups was the cost for hospitalisation due to causes not related to MI, angina or chronic heart failure (p = 0.0043), resulting in higher hospitalisation ($6,841 vs. $4,984, p = 0.036) and total treatment cost ($10,574 versus $8,534, p = 0.021) for patients randomised to receive HC versus UC. Analyses using completed cases only did not result in statistically significant higher costs for HC compared to UC ($9,677 versus $7,152, p = 0.124).

### Cost effectiveness

Within the six-months trial duration, the incremental cost was $2,040 and the incremental effectiveness was 0.012 QALYs (95% CI: -0.016 to 0.040). The incremental cost-effectiveness ratio (ICER) of HC compared to UC for patients with a recent MI was $85,423/QALY (95% CI: $25,327, dominated).

## Discussion

As patients with CHD have a high risk of a secondary event, there is a need for effective secondary prevention interventions with good uptake. This study examined the cost-effectiveness of a novel telephone-delivered secondary prevention program for MI patients. We found a significant improvement in health status as assessed with the SF-6D in both the HC and UC groups at six and 12 months, although the difference between groups was not significant. The intervention was also associated with higher costs compared to UC. This higher cost was mainly driven by higher non-cardiovascular hospitalisation (e.g. renal dialysis, urinary tract and renal disorders, cancer and gastric disorders) rather than the costs of running the intervention.

The primary outcomes paper reported that the intervention resulted in a significant positive effect on mental HRQoL, as well as Social Functioning and Role Emotional subscales of the SF-36 compared with UC [16]. Patients were also more likely to meet recommended levels of physical activity, BMI, vegetable intake and alcohol consumption. Using the SF-6D summary score, we did not observe an intervention effect on utility at 6 or 12 months. This could be a result of the fact that patients in the HC group had numerically lower scores for physical HRQoL, which may result in no differences on an overall score. It should be noted that UK weights were used for the SF-6D, as no Australian weights are available.

There is very limited data available on the cost-effectiveness of telephone intervention programs as a secondary intervention for patients with following MI. Telephone delivered interventions consist of telephone calls from a health professional who provides health advice (coaching) to encourage and support behavioural changes. In contrast, telemonitoring programs involve patients using monitoring devices to measure certain clinical parameters (e.g. blood glucose levels) of which the results are then transmitted by telephone to the health provider. As such telemonitoring programs are not easily comparable with programs focusing more on behavioural changes (i.e. telephone delivered interventions). A recent Cochrane review included structured telephone interviews or telemonitoring for patients with chronic heart failure, with the latter being less relevant [4]. Nine studies included costing of structured telephone support programs; however, the information available for those studies was limited, with often only information on the cost of the program. As our program was undertaken by a health coach, the costs of each session is relatively low, compared to fully integrated telehealth programs. The cost of the health coaching sessions ($37 per session) accounted for less that 4% of the overall cost for the HC group.

The increased cost for hospitalisation due to causes other than CHD events in the HC group could be due to better education, support and monitoring in this group which may have lead to seeking medical intervention earlier in a disease process. This could potentially result in cost-savings in the longer term. As we have analysed health care utilisation at six months, the longer term health care utilisation and costs is unclear.

Telephonic disease management was not effective and was not cost-effective in a randomised trial of patients with systolic or diastolic heart failure over 18 months [21]. That program improved overall survival; however, the disease management program was costly and did not reduce health service utilisation. Telephone delivered intervention for physical activity and diet in a group of adults with chronic disease was considered cost-effective at an ICER of $12,153/QALY for telephone counselling compared to real-life care [14]; however that study used a theoretical UC group instead of the group of randomised controls [14]. In another clinical trial comparing telephone counselling with UC reported an ICER of $78,489/QALY [22]. Other telephone-intervention studies have reported cost-savings from reduced readmission rates, reduced length of stay [23] whereas another study reported an unacceptably high cost per QALY gained of $146,870 [21]. The large variance in cost-effectiveness results has provided impetus, in part, for a large-scale telephone intervention study of health coaching with >45,000 patients [24].

In the present study there were losses to follow-up and incomplete data; therefore multiple imputation techniques were applied. For hospitalisation rates and costs, data was available for all patients. Therefore, a comparison for the number of hospitalisations between the completed cases and multiple imputation analyses (including all patients) can give an indication of the health of the patients who completed and did not complete the six-month follow-up. Patients who continued follow-up had fewer hospitalisations compared to patients who had missing health services data. No differences in HRQoL were observed between patients with and without missing data, using multiple imputation techniques. While missing data is potentially a limitation of the study, hospitalisation data in public hospitals was available for all patients randomised in the study and the hospitalisation costs accounted for the majority of the difference in cost between the two treatment groups (91% for multiple imputation analysis).

This analysis relied upon self-reported patient data; this leads to some uncertainty around the accuracy of the data. Self-reporting can underestimate the number of GP visits using longer recall periods (one year) compared to one month recall period [25]. We performed an analysis on a subsample of 10% to check patient reported data with claims made by their physician. There was significant underreporting for the six month recall period; however, there was a reasonable correlation between number of reported GP visits and claims made by their GP. As such, linear regression methodology was applied. As we could not verify the self-reported data for visits to specialists, other health professionals, health services and medication use with claims data, the self-reported data has been used without any modification. It is uncertain whether the use of self-reported data would bias the results in favour of health coaching. Hospitalisation data for each patient was available for all public hospital admissions in Queensland; however, some patients reported an admission to hospital in a private hospitals or public hospitals in a different state. Self-reported admission to private hospitals was not different between the two study groups, and therefore no adjustments were performed.

## Conclusions

ProActive Heart, a telephone delivered CHD secondary prevention program, was not a cost-effective intervention in the short-term compared to UC. There was no significant improvement in utility and it resulted in significantly increased costs. However, while we have not assessed this in the current study, higher cost may result in future cost-savings as patients are potentially better monitored, and therefore it could be suggested that health problems may be identified at an earlier stage resulting in better health outcomes.

## Competing interests

The authors have no competing interest to disclose.

## Authors’ contributions

ET designed and performed all statistical analyses and drafted the manuscript. ALH and BO developed the study concept, aims and initiated the project. ALH oversaw the implementation of the trial and the collection of data. PAS developed the economic evaluations. All authors read and approved the final manuscript.

## Pre-publication history

The pre-publication history for this paper can be accessed here:

http://www.biomedcentral.com/1471-2261/13/33/prepub

## Supplementary Material

Additional file 1 Additional files for web appendix. Click here for file
