# Supplementary material for: Cost-effectiveness of a coronary heart disease secondary prevention program in patients with myocardial infarction: results from a randomised controlled trial (ProActive Heart)
Source: BMC Cardiovasc Disord. 2013 May 1;13:33. doi: 10.1186/1471-2261-13-33 (PMC3646683; doi:10.1186/1471-2261-13-33)
Supplement: Additional file 1 — Additional files for web appendix. [file 1471-2261-13-33-S1.doc]

# Additional files for web appendix

### Web Appendix Table 1: Costs of Health Care Resources

| **Resource use** | **Utilisation** | **Cost (A$)** | **Source** |
| --- | --- | --- | --- |
| **HC session** | Health Coach | $37 | ProActive Heart trial |
| **GP visit** | Validation of subsample ^a^ | $39.92 | MBS weighted price from subsample analyses |
| **Specialist visit** | Self-reported | | |
| First visit  Subsequent visit |  | $79.05  $39.70 | MBS item 104  MBS item 105 |
| **Other health professionals** | Self-reported | | |
| First 5 visits  Additional visits |  | $57.55  $18.40 | MBS items 10953/10960/10951/10968/10962/10968  MBS items 81115/81105/81125 |
| **Health services** | Self-reported | | |
| *Prince Charles & Royal Brisbane and Women’s Hospital* | | | |
| Outpatient |  | $236.31 | National Hospital Cost data Collection, Cost Report Round 12 (2007-2008) |
| Day procedures |  | $725.32 |
| Emergency |  | $465.68 |
| *Other hospitals* | | |
| Outpatient |  | $236.31 |
| Day procedures |  | $532.89 |
| Emergency |  | $257.43 |
| **Medication** | Self-reported |  | PBS Schedule (November 2008 prices), assumption that patient will take medication for six months. |
| **Hospital admittance** | Queensland Health Admitted Patient Data Collection |  | Cost weight ICD-10 and AR-DRG codes version 5.0 (2005-2006) inflation applied |

^a^ To adjust for underreported a correlation coefficient of Y=1.067x + 4.149 (r = 0.592, p = 0.000) was applied to the patient reported GP visits to estimate the actual GP visits.

### Web Appendix Table 2: Baseline characteristics comparison all patients and completed cases at 6 months.

|  | ***All patients*** | | | ***Completed case*** | | |
| --- | --- | --- | --- | --- | --- | --- |
|  | **HC** | **HC** | ***P-value*** | **HC** | **UC** | ***P-value*** |
| N | **136** | **136** |  | **136** | **146** |  |
| Age, year | 62.0 (11.0) | 62.0 (11.0) | 0.212 | 62.0 (11.0) | 59.5 (10.6) | 0.059 |
| Male, % | 77.2% | 77.2% | 0.579 | 77.2% | 78.1% | 0.860 |
| Smoking status   - never smoked - previous smoker - current smoker | 25.1%  43.7%  31.1% | 30.2%  38.1%  31.6% | 0.398 | 26.5%  44.1%  29.4% | 27.4%  39.0%  33.6% | 0.656 |

### Web Appendix Table 3: SF-6D index at baseline and change between health coaching and usual care – completed cases

|  | **HC**  **mean (SE)** | **UC**  **mean (SE)** | **mean difference**  **(95% CI)** | ***p-value*** |
| --- | --- | --- | --- | --- |
| **6 month data** | | | | |
| **N** | 141 | 153 |  |  |
| **Baseline** | 0.672 (0.011) | 0.675 (0.011) | -0.003 (-0.034, 0.028) | 0.766 |
| **Change from baseline ^a^** | 0.141 (0.012) | 0.137 (0.012) | 0.004 (-0.029, 0.037) | 0.698 |
| **12 month data** | | | | |
| **N** | 118 | 124 |  |  |
| **Baseline** | 0.683 (0.012) | 0.680 (0.013) | 0.002 (-0.032, 0.037) | 0.818 |
| **Change from baseline ^a^** | 0.142 (0.014) | 0.136 (0.014) | 0.006 (-0.033, 0.046) | 0.574 |

^a^ adjusted for baseline value

**Web Appendix Table 4: Utilisation and cost of health care services – completed cases**

|  | **Utilisation** | | | **Cost** | | |
| --- | --- | --- | --- | --- | --- | --- |
| **Mean (se)** | **HC** | **UC** | **p-value** | **HC** | **UC** | ***p-value*** |
| N | 136 | 146 |  | 136 | 146 |  |
| Health coach sessions | 8.2 (0.2) |  | N/A | $305 ($6) |  | N/A |
| GP visits | 9.9 (0.4) | 10.9 (0.4) | 0.153 | $395 ($17) | $434 ($16) | 0.153 |
| Specialist visits | 1.4 (0.2) | 0.7 (0.1) | **0.001** | $75 ($9) | $42 ($6) | **0.001** |
| Other health professionals | 1.4 (0.3) | 1.0 (0.2) | 0.074 | $64 ($11) | $44 ($9) | 0.074 |
| Health services | 3.4 (0.5) | 4.4 (0.7) | 0.134 | $824 ($125) | $1,062 ($156) | 0.117 |
| Medication   - cardiac system - lipid modifying drugs - antithrombotic agent - drugs used in diabetes - other medicines | 2.3 (0.1)  0.8 (0.0)  1.6 (0.0)  0.2 (0.1)  1.2 (0.1) | 2.3 (0.1)  0.9 (0.0)  1.7 (0.1)  0.2 (0.0)  1.1 (0.1) | 0.994  0.372  0.448  0.977  0.627 | $296 ($24)  $476 ($21)  $385 ($20)  $112 ($42)  $235 ($35) | $314 ($22)  $503 ($21)  $396 ($21)  $112 ($38)  $294 ($75) | 0.341  0.467  0.586  0.910  0.713 |
| Hospital admittance/patient   - MI/angina/CHF - other cause - total | 0.5 (0.1)  0.4 (0.2)  1.0 (0.2) | 0.4 (0.1)  0.1 (0.0)  0.5 (0.1) | 0.605  **0.023**  0.116 | $4,581 ($833)  $1,928 ($760)  $6,509 ($1,095) | $3,573 ($693)  $378 ($150)  $3,951 ($714) | 0.555  **0.022**  0.096 |
| Total cost |  |  |  | $9,677 ($1,118) | $7,152 ($729) | 0.124 |

N/A = not applicable; MI = myocardial infarction; CHF = chronic heart failure; HC = health coaching; UC = usual care
